# The causal relationship between COVID-19 and ten esophageal diseases: a study utilizing Mendelian randomization

**DOI:** 10.3389/fmed.2024.1346888

**Published:** 2024-05-01

**Authors:** Xu He, Yue Li, Jun Liu, Guanqiang Yan, Xiang Gao, Guosheng Li, Longqian Wei, Guiyu Feng, Jingxiao Li, Huafu Zhou

**Affiliations:** Department of Cardio-Thoracic Surgery, The First Affiliated Hospital of Guangxi Medical University, Nanning, China

**Keywords:** causal relationship, COVID-19, esophageal diseases, Mendelian randomization, SARS-CoV-2

## Abstract

**Background:**

Clinical signs of dysphagia, pancreatic achalasia, and esophagitis have been reported in patients with COVID-19. However, the causal relationship between COVID-19 and esophageal diseases is not clear. Therefore, we utilized Mendelian randomization to explore the potential association between COVID-19 and esophageal diseases.

**Methods:**

The summary statistics for a Genome-wide association study (GWAS) were obtained from The COVID-19 Host Genetics Initiative, encompassing four types of COVID-19 as exposure: severe COVID-19, hospitalized COVID-19 versus ambulatory COVID-19, hospitalized COVID-19 versus uninfected, and confirmed COVID-19. Additionally, summary statistics for ten esophageal diseases as outcomes were sourced from the GWAS Catalog and FinnGen databases. Univariate Mendelian randomization (MR) analysis was utilized to thoroughly investigate and validate the potential causal association between COVID-19 and various esophageal conditions, including esophageal varices, Barrett’s esophagus, esophagitis, esophageal obstruction, esophageal ulcer, esophageal perforation, gastroesophageal reflux, congenital esophageal malformations, benign esophageal tumors, and esophageal adenocarcinoma.

**Results:**

An inverse variance-weighted (IVW) model was utilized for univariate Mendelian randomization (MR) analysis, which revealed that genetic liability in patients with confirmed COVID-19 was associated with esophageal obstruction (OR [95% CI]: 0.5275458 [0.2822400–0.9860563]; *p*-value = 0.0450699). Furthermore, a suggestive causal association was found between genetic liability and a reduced risk of benign esophageal tumors (OR [95% CI]: 0.2715453 [0.09368493–0.7870724]; *p*-value = 0.0163510), but with a suggestively increased risk of congenital esophageal malformations (OR [95% CI]: 6.959561 [1.1955828–40.51204]; *p*-value = 0.03086835). Additionally, genetic liability in hospitalized COVID-19 patients, compared to non-hospitalized COVID-19 patients, was suggestively associated with an increased risk of esophagitis (OR [95% CI]: 1.443859 [1.0890568–1.914252]; *p*-value = 0.01068201). The reliability of these causal findings is supported by Cochran’s Q statistic and the MR-Egger intercept test.

**Conclusion:**

The results of this study suggest the existence of a causal relationship between COVID-19 and esophageal diseases, highlighting differing risk effects of COVID-19 on distinct esophageal conditions.

## 1 Introduction

According to data from the World Health Organization (WHO) as of November 2023, the 2019 coronavirus disease (COVID-19) has resulted in over 770 million Severe Acute Respiratory Syndrome Coronavirus 2 (SARS-COV-2) infections and more than 6.9 million deaths (Available online: https://covid19.who.int/). Unlike Middle East respiratory syndrome coronavirus (MERS-CoV) and seven other human coronaviruses (HCoVs), SARS-COV-2 exhibits genetic alterations, including the D614G mutation in the S1 subunit of the surface spiking protein and a modification in the Flynn protease cleavage site. These genetic changes notably contribute to the increased transmissibility of SARS-COV-2 and are associated with a spectrum of respiratory, gastrointestinal, and neurological manifestations, encompassing symptoms such as fever, cough, abdominal pain, diarrhea, as well as anosmia and ageusia. Clinical observations reveal that a significant proportion of SARS-COV-2 patients (80–90%) either remain asymptomatic or experience mild symptoms, thereby directing the treatment approach towards antiviral interventions such as antiviral drugs, neutralizing antibody therapy, Janus kinase inhibitors, and steroids (1–4), however, according to a retrospective study covering a 2- to 6-month recovery period from SARS-COV-2 infection, some patients were found to experience persistent disturbances in smell and taste, increased fatigue, chest pains, muscle pains, cognitive symptoms, and abdominal symptoms. Additionally, these individuals were observed to be at a higher risk of developing anxiety disorders, dementia, and Parkinson’s syndrome (5–7). Therefore, it is important to investigate whether COVID-19 has an effect on other systems in the body and to provide guidance for prevention and the development of effective therapeutic strategies.

In modern times, with the continuous improvement of living standards, the prevalence of smoking, alcohol consumption, and excessive nitrosamine intake among the population has increased. As a result, there has been a rise in the proportion of individuals affected by various esophageal diseases, including Barrett’s esophagus, esophagitis, esophageal ulcers, gastro-esophageal reflux disease, esophageal cancer, and other related disorders. This upward trend has been observed to occur annually (8–13). Additionally, in the context of the global SARS-COV-2 pandemic, there have been reports indicating that SARS-COV-2 can cause dysphagia, pancreatic achalasia, and esophagitis. Pertinently, it has been observed that SARS-COV-2 is capable of infecting esophageal cells through the expression of ACE2 and TMPRSS2, potentially contributing to the development of esophageal carcinogenesis (14–17). However, due to the current decrease in the number of diagnosed SARS-COV-2 patients (18), the definitive impact of SARS-COV-2 on various facets of esophageal disease remains elusive, and there is a lack of unequivocal studies establishing a causal link between COVID-19 and esophageal disease. Thus, it is valuable to investigate the potential causal relationship between SARS-COV-2 and various esophageal conditions, such as esophageal varices, esophagitis, Barrett’s esophagus, esophageal obstruction, esophageal ulcers, esophageal perforation, gastroesophageal reflux disease, congenital esophageal malformations, esophageal adenocarcinomas, and benign esophageal tumors. These investigations are of utmost importance for protecting the esophageal safety of individuals infected with SARS-COV-2.

In contrast to the causal conclusions drawn in retrospective studies, Mendelian randomization using single nucleotide polymorphisms (SNPs) as an instrumental variable can accurately reflect the causal relationship between exposures and outcomes. This is achieved through Mendelian randomization analyses that satisfy the criteria of independence, association, and exclusivity, effectively reducing the effects brought about by genetic variants and other confounding factors (19). In summary, the purpose of this paper is to investigate the potential causal relationship among critically ill COVID-19 patients, COVID-19 hospitalized and COVID-19 ambulatory patients, COVID-19 hospitalized patients and diagnosed COVID-19 patients, and ten distinct types of esophageal diseases. These diseases include esophageal varices, esophagitis, Barrett’s esophagus, esophageal obstruction, esophageal ulcers, esophageal perforation, gastroesophageal reflux disease, congenital esophageal malformations, esophageal adenocarcinomas, and benign esophageal tumors. This investigation will be conducted through the use of MR analysis. The ultimate objective is to provide valuable insights that can guide the prevention and treatment of esophageal diseases following infection with SARS-COV-2.

## 2 Methods

### 2.1 Research design

**Figure 1:**
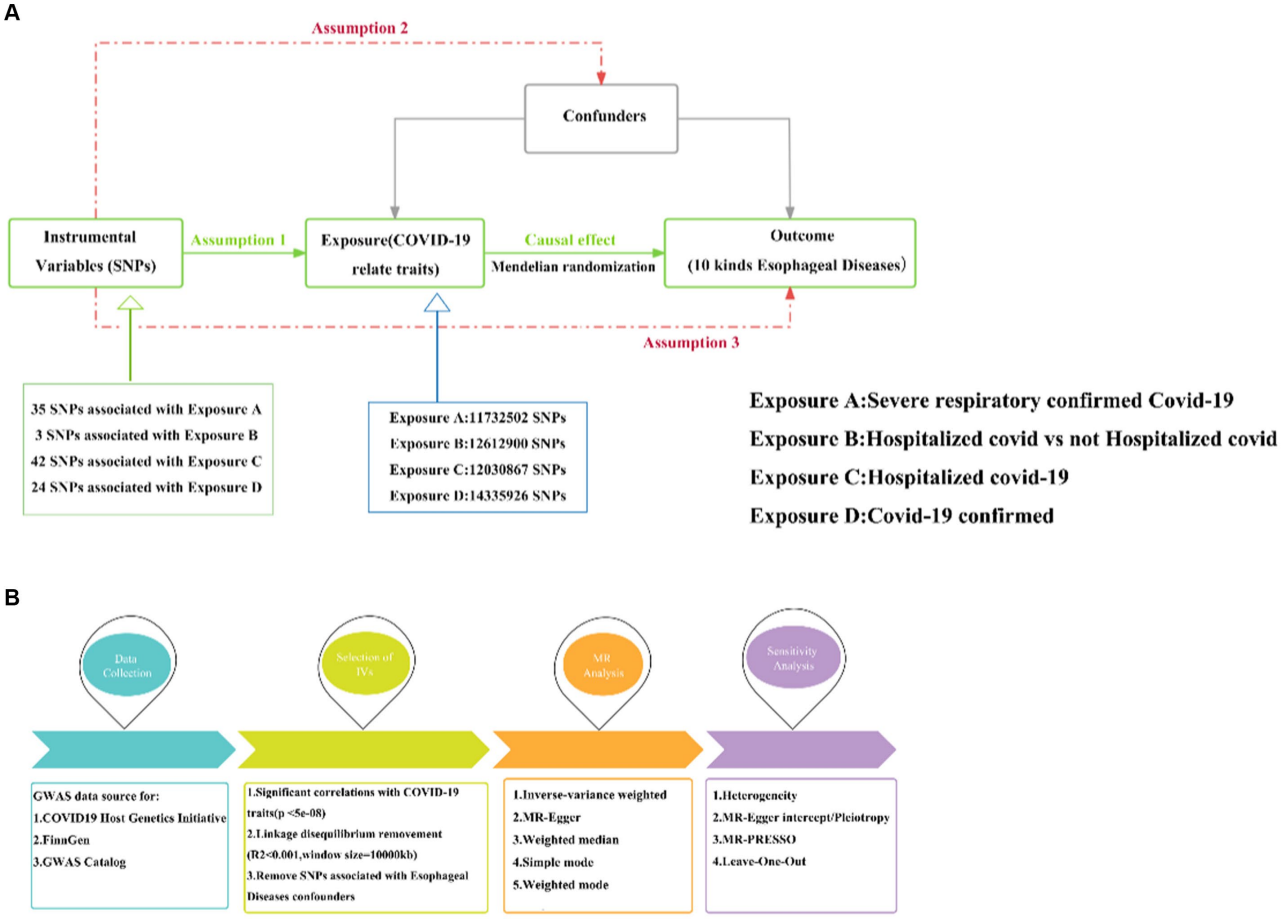
MR research design. **(A)** Three hypotheses for MR. **(B)** Basic flow of MR study between COVID-19 and esophageal diseases. MR, Mendelian randomization; COVID-19, Coronavirus disease 2019; Assumption 1: Assumption of association; Assumption 2: Assumption of independence; Assumption 3: Assumption of exclusivity.

In this study, we utilized the most effective genetic variation tool, known as the instrumental variable (IV), to investigate the causal relationship between COVID-19 and ten esophageal diseases. The analysis was conducted based on the principles of two-sample MR. According to MR theory, a valid IV must satisfy three key assumptions, which are essential for ensuring the validity of subsequent analyses (20):

1. The IVs need to correlate strongly with relevant features of COVID-19 as an exposure.
2. The IVs are not associated with other potential confounders affecting COVID-19 and esophageal disease.
3. The IVs are not directly associated with esophageal disease as an outcome, but only indirectly through exposure (see Figure 1).

### 2.2 Sources of the GWAS dataset

We obtained four types of GWAS datasets related to COVID-19 as an exposure from The COVID-19 Host Genetics Initiative,^1^ including critically ill COVID-19, hospitalized COVID-19 versus non-hospitalized COVID-19, hospitalized COVID-19 versus uninfected individuals, and confirmed COVID-19 cases. Additionally, GWAS data on esophageal adenocarcinoma were obtained from the GWAS Catalog,^2^ and the remaining nine types of esophageal diseases were sourced from FinnGen’s R8 dataset.^3^ Table 1 presents the sample information for COVID-19 alongside the ten types of esophageal diseases.

**Table 1:** Information on GWAS datasets for esophageal diseases and COVID-19 traits.

Outcome/exposure	Cases	Control	Primary population	Data source
Barrett esophagus	946	292,256	European	FINNGEN
Perforation of esophagus	103	292,256	European	
Oesophageal obstruction	879	292,256	European	
Esophageal varices	894	295,014	European	
Esophageal ulcer	1840	292,256	European	
Benign esophageal tumors	260	342,239	European	
Esophagitis	1,318	292,256	European	
Gastroesophageal-reflux disease	22,867	292,256	European	
Congenital-esophageal malformation	94	341,501	European	
Esophageal adenocarcinoma	4,112	17,159	European	GWAS Catalog
**Covid-19 traits**				
Severe respiratory confirmed Covid-19	18,152	1,145,546	European	The COVID-19 Host Genetics Initiative
Hospitalized covid vs. not hospitalized covid	16,512	71,321	European	
Hospitalized covid-19	44,986	2,356,386	European	
Covid-19 confirmed	159,840	2,782,977	European	

COVID-19, Coronavirus disease 2019; GWAS, Genome wide association study.

### 2.3 Instrumental variables (IVs) selection

In order to ensure a strong correlation with COVID-19, we carefully selected single nucleotide polymorphisms (SNPs) as instrumental variables (IVs) based on the criterion of *p* < 5 × 10^–8.^ Subsequently, thresholds of *r*^2^ < 0.001 and Kb = 10,000 were applied to eliminate any effects of linkage disequilibrium (LD) reflections. Additionally, palindromic SNPs were excluded from the analysis. To establish independence between the instrumental variables and COVID-19, we utilized the PhenoScannerV2 database^4^ to identify and remove potential confounding factors that could impact both exposure and outcome. This meticulous approach ensured the integrity of the IVs and their association with COVID-19 (21).

### 2.4 MR analysis

In conducting the two-sample Mendelian randomization (MR) analysis comparing COVID-19 and esophageal disease, we utilized a total of five MR methods: inverse variance weighted (IVW), MR Egger, weighted median, simple mode, and weighted mode. These methods were employed to systematically investigate the potential causal relationship between the exposure and the outcome. Notably, the IVW method analyses the relationship of causal effects arising from multiple SNPs without considering the intercept and fits the results with the inverse of the variance of the endpoints as weights (22). In contrast, MR-Egger enables causal analysis through the examination of the slope coefficient, thereby providing an alternative avenue for assessing causality. Conversely, the weighted median method showcases its capacity to compute unbiased estimates, even in the presence of a significant fraction of invalid instrumental variables (IVs) (23–25). Therefore, given the prevalent use of the IVW in two-sample Mendelian randomization studies, we have utilized IVW as the primary analytical approach, with the remaining four methods serving as supplementary tools to enhance the findings derived from IVW.

### 2.5 Sensitivity analysis

To ensure the stability of the examined causal relationship between COVID-19 and esophageal diseases, we undertook heterogeneity analysis using Cochran’s Q statistic. A *p*-value greater than 0.05 indicated the absence of significant heterogeneity. Additionally, we employed MR pleiotropy residual sum and outlier (MR-PRESSO) to detect any outlier values. In the context of horizontal pleiotropy, we conducted MR-Egger intercept analysis and implemented leave-one-out analysis to assess whether a single SNP influenced the causal relationship between exposure and outcome. A *p*-value exceeding 0.05 suggested no significant presence of horizontal pleiotropy.

### 2.6 Statistical analysis

The analyses were conducted using the “TwoSampleMR (v0.5.7)” and “data.table” package within R software (v4.3.1). In the context of two-sample MR analyses, corrected multiple testing was implemented utilizing the Bonferroni method. Significance for causality was defined as a *p*-value less than 1.25 × 10^−4^ (0.05/40, accounting for 4 exposures and 10 outcomes), while *p*-values falling between 1.25 × 10^−4^ and 0.05 were considered indicative of suggestive causality.

## 3 Results

### 3.1 IVs for COVID-19

There were 35, 3, 42, and 24 IVs in severe COVID-19 patients, hospitalized COVID-19 patients vs. non-hospitalized COVID-19 patients, hospitalized COVID-19 patients vs. the general population, and confirmed COVID-19 patients, after calculating by the cascade imbalance effect (Supplementary Table S1).

### 3.2 Causal effect of severe COVID-19 on esophageal disease

Our findings revealed that a genetic liabilities to severe COVID-19 was potentially associated with an increased risk of congenital esophageal malformations, indicating a trend towards a causal relationship. However, this association did not reach statistical significance (OR [95% CI]: 1.4425145 [0.9545951–2.179823]; *p*-value = 0.08196500). Conversely, for the other nine esophageal disorders examined, no causal association was observed between severe COVID-19 and these conditions (Supplementary Table S2).

### 3.3 Causal effect of hospitalized versus non-hospitalized COVID-19 on esophageal disease

**Figure 2:**
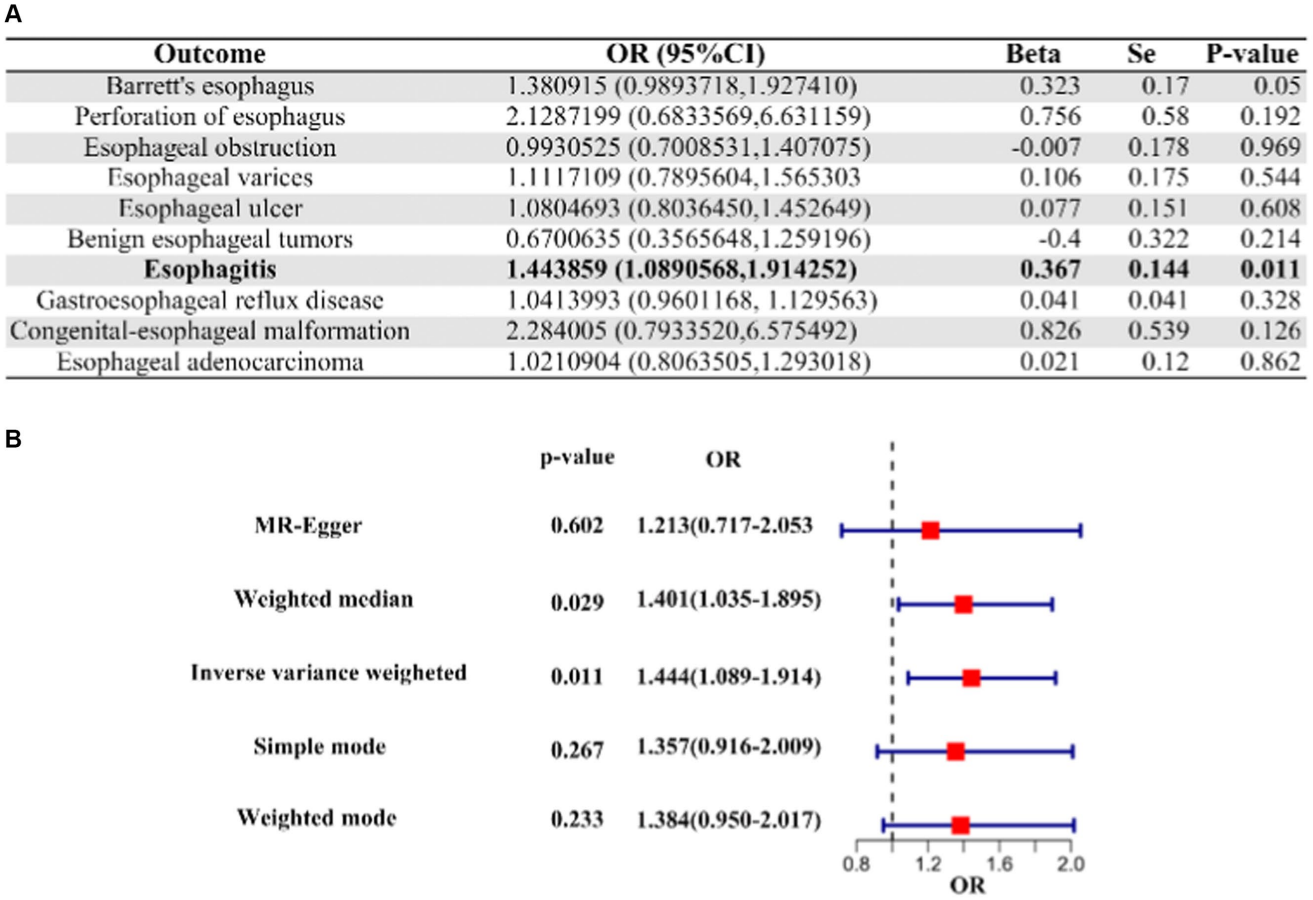
IVW Mendelian randomization analysis and forest plot of hospitalized COVID-19 versus non-hospitalized COVID-19 on the risk of esophageal disease. **(A)** Results of IVW analysis for 10 esophageal diseases. **(B)** Results of the analysis of 5 MR methods for oesophagitis. IVW, inverse variance weighting; CI, confidence interval; COVID-19, coronavirus disease in 2019; OR, odds ratio.

Our analysis about COVID-19 patients who underwent hospitalization versus those who did not revealed a suggestive causal association between their genetic liabilities and an increased risk of esophagitis (OR [95% CI]: 1.443859 [1.0890568–1.914252]; *p*-value = 0.01068201; Figure 2). However, it is important to note that COVID-19 did not demonstrate a causal relationship with other esophageal diseases in the context of comparisons between hospitalized and non-hospitalized patients (Figure 2, Supplementary Table S2).

### 3.4 Causal effect of hospitalized COVID-19 on esophageal disease

Upon comparing hospitalized COVID-19 patients with the uninfected general population, we observed a trend suggesting a potential causal relationship between genetic liabilities for hospitalization and an elevated risk of congenital esophageal malformations, although this finding did not reach statistical significance (OR [95% CI]: 1.7542346 [0.9697958–3.173182]; *p*-value: 0.06308687). Furthermore, we did not identify any causal association between COVID-19-related hospitalization and other esophageal diseases (Supplementary Table S2).

### 3.5 Causal effects of COVID-19 on esophageal diseases

**Figure 3:**
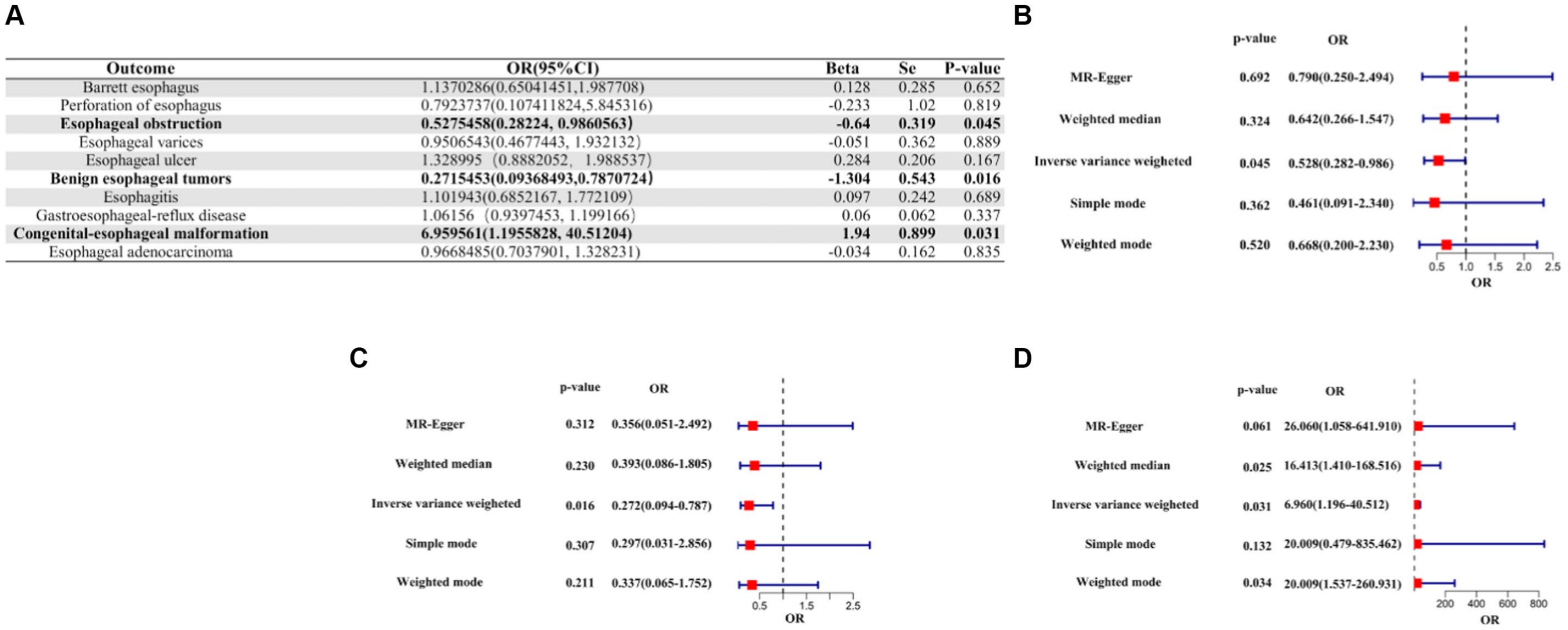
IVW Mendelian randomization analysis of COVID-19 on the risk of esophageal diseases. **(A)**. Results of the IVW analysis of confirmed COVID-19 on the risk of 10 esophageal diseases. **(B)** Forest plot of MR analysis of confirmed COVID-19 on esophageal obstruction. **(C)** Forest plot of MR analysis of confirmed COVID-19 for benign esophageal tumors. **(D)** Forest plot of MR analysis of confirmed COVID-19 for congenital esophageal malformations. MR, Mendelian randomization; IVW, inverse variance weighted; CI, confidence interval; COVID-19, coronavirus disease 2019; OR, odds ratio.

A suggestive causal association was observed between genetic liabilities for COVID-19 and a reduced risk of esophageal obstruction (OR [95% CI]: 0.5275458 [0.2822400–0.9860563]; *p*-value = 0.0450699), as well as for a reduced risk of benign esophageal tumors (OR [95% CI]: 0.2715453 [0.09368493–0.7870724]; *p*-value = 0.0163510). Conversely, genetic liability for COVID-19 was linked to an increased risk of congenital esophageal malformations (OR [95%CI]: 6.959561 [1.1955828–40.51204]; *p*-value = 0.03086835), suggesting a causal relationship. Notably, no causality was observed between COVID-19 and the other seven esophageal diseases, except for the three mentioned above (Figure 3, Supplementary Table S2).

### 3.6 Sensitivity analysis

The results of the sensitivity analysis confirmed the robustness of the aforementioned causality, demonstrating a consistent trend towards causation. The heterogeneity test revealed heterogeneity only in the case of COVID-19 severe infection and esophageal adenocarcinoma within the MR analysis (Cochran’s Q statistic, with *p*-value >0.05 indicating the absence of heterogeneity; Supplementary Table S3). A scatterplot was presented to illustrate the causal effect of COVID-19 on esophagitis, esophageal obstruction, benign esophageal tumors, and congenital esophageal malformations using three different methods: IVW, MR-Egger, and weighted-median approaches. A positive correlation was represented by slopes greater than zero, while slopes less than zero indicated a negative correlation (Supplementary Figure S1).

**Figure 4:**
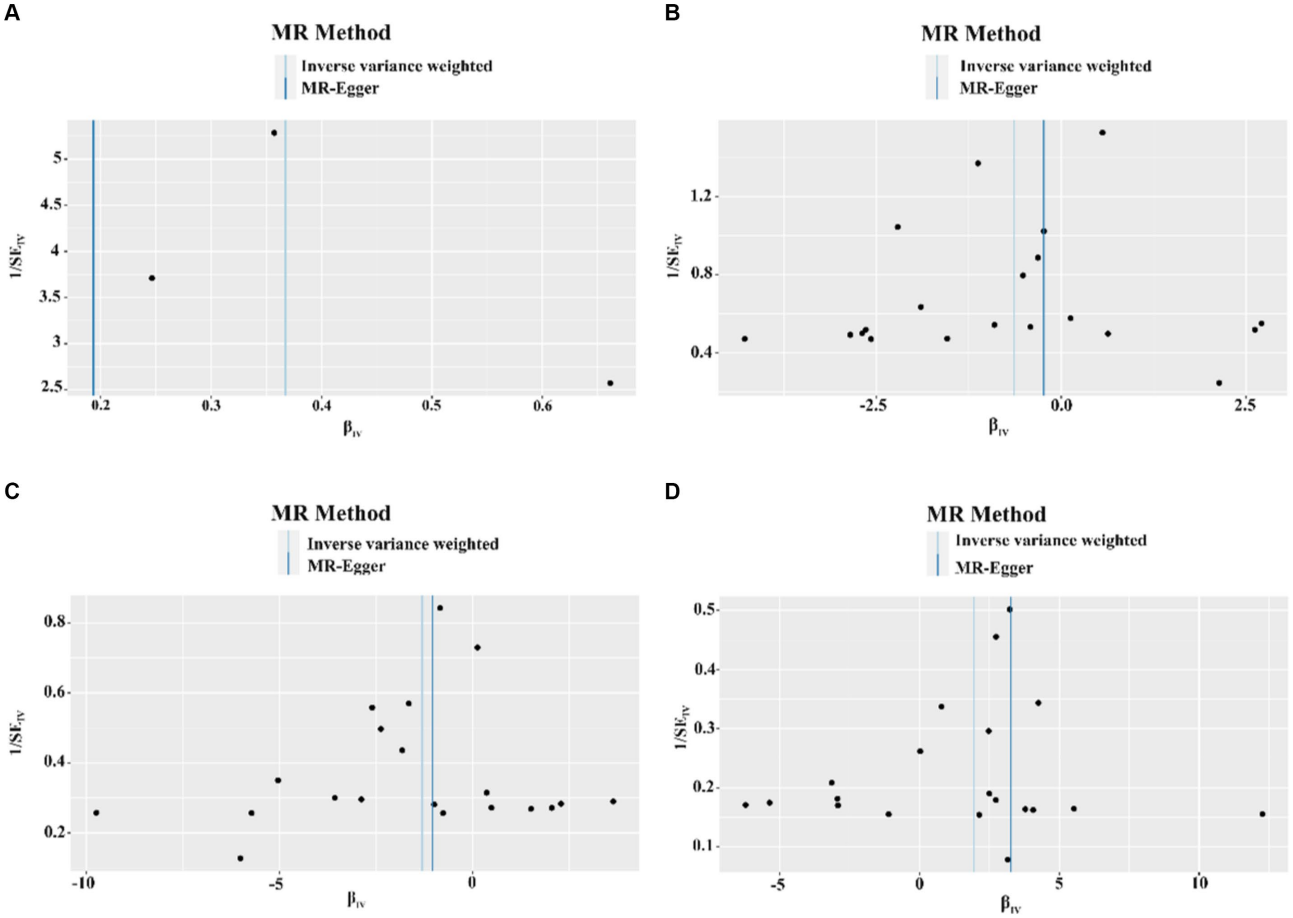
Funnel plot of causal effect of COVID-19 on esophageal disease. **(A)** Funnel plot of causal effect of hospitalized COVID-19 versus non-hospitalized COVID-19 on esophagitis. **(B)** Funnel plot of causal effect of confirmed COVID-19 on esophageal obstruction. **(C)** Funnel plot of causal effect of confirmed COVID-19 on benign esophageal tumors. **(D)** Funnel plot of the causal effect of COVID-19 on congenital esophageal obstruction.

**Figure 5:**
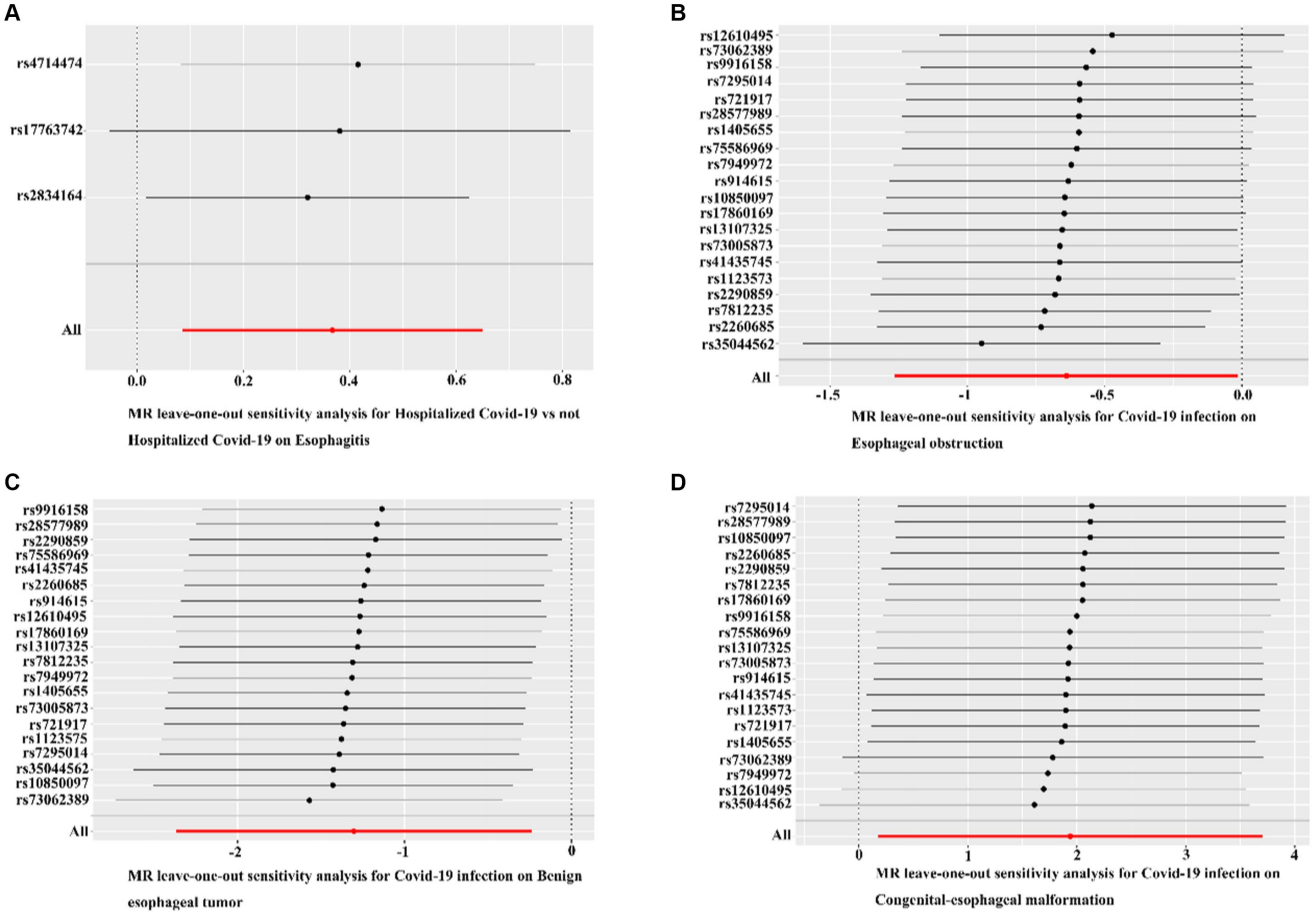
Leave-one-out analyses for COVID-19 on esophageal diseases. **(A)** Leave-one-out analyses for hospitalized COVID-19 versus non-hospitalized COVID-19 with esophagitis. **(B)** Leave-one-out analysis of confirmed COVID-19 versus esophageal obstruction. **(C)** Leave-one-out analysis of confirmed COVID-19 with benign esophageal tumor. **(D)** Leave-one-out analysis of confirmed COVID-19 with congenital esophageal malformation. Black dots indicate that individual SNP causal effects were assessed using the IVW method; red dots indicate inverse variance weighted estimates using all SNPs.

The MR-Egger regression results did not provide evidence of significant pleiotropy at a *p*-value >0.05. Additionally, the MR-PRESSO global test outcomes indicated the absence of significant outliers that could contribute to a causal effect (*p*-value >0.05). The symmetry observed in the funnel plot suggested no significant heterogeneity (Figure 4). Furthermore, the results of the Leave-one-out analysis demonstrated that no individual single nucleotide polymorphism (SNP) exerted a dominant influence on the causal effects observed between the related outcomes (Figure 5).

## 4 Discussion

With the onset of the global COVID-19 pandemic, an increasing number of patients are clinically diagnosed with symptoms extending beyond the respiratory system. This can be attributed to the structural composition of the spike (S) protein of the SARS-CoV-2 virus, which comprises a lengthy N-terminal S1 subunit and a shorter C-terminal S2 subunit. These subunits serve as the primary functional components of the protein, with the S1 subunit predominantly engaging with the angiotensin-converting enzyme 2 (ACE2) receptor, which exhibits widespread expression across various tissues and organs of the human body. The interaction between the S1 subunit and the ACE2 receptor necessitates the activation of a serine protease known as TMPRSS2. This protease plays a crucial role in cleaving the S1 and S2 subunits of the S protein, thereby facilitating the binding of the separated S2 subunit to the host cells. Consequently, this binding allows for the entry of the SARS-CoV-2 virus into the host cells, subsequently triggering the release of replication-related substances within these cells. As a result, the virus further propagates and inflicts damage upon adjacent cells, perpetuating the cycle of infection and cellular injury (26–30). However, As the number of newly detected cases gradually decreases, it becomes increasingly challenging to ascertain the long-term effects of COVID-19 on a wide spectrum of diseases. Research has indicated that SARS-COV-2 can invade esophageal epithelial cells via the ACE2 receptor (31). Therefore, for the first time, we have used MR to explore the potential causal relationship between COVID-19 and esophageal diseases. This investigation aims to facilitate subsequent rehabilitation efforts for COVID-19-induced esophageal disorders.

Our research findings indicate potential causal links between COVID-19 and four esophageal diseases: esophagitis, esophageal obstruction, benign esophageal tumor, and congenital esophageal malformation. We found no evidence of genetic pleiotropy at the gene level of COVID-19 as an exposure to these esophageal diseases. Furthermore, there was no causal effect resulting from the presence of a single SNP driver, and no heterogeneity factors were found to influence the results. Our sensitivity analyses confirmed the robustness of these findings. We observed that genetic liability was associated with an increased risk of esophagitis in hospitalized COVID-19 patients compared to non-hospitalized COVID-19 patients (odds ratio [OR] [95% confidence interval]: 1.443859 [1.0890568–1.914252]; *p*-value = 0.01068201). Conversely, genetic liability was associated with a six-fold increased risk of congenital esophageal malformations in COVID-19 patients with SARS-CoV-2 infection (OR [95% CI]: 6.959561 [1.1955828–40.51204]; *p*-value = 0.03086835). Surprisingly, SARS-CoV-2 infection reduced the risk of esophageal obstruction by almost 50% (OR [95% CI]: 0.5275458 [0.2822400–0.9860563]; *p*-value = 0.0450699), and by as much as 72% for benign esophageal tumors (OR [95% CI]: 0.2715453 [0.09368493–0.7870724]; *p*-value = 0.0163510). However, there was a trend towards a significantly lower risk of congenital esophageal malformations for severe COVID-19 compared to mild COVID-19 (OR [95% CI]: 1.4425145 [0.9545951–2.179823]; *p*-value = 0.08196500). While these results did not reach statistical significance for the characteristics of the four types of COVID-19, they provide suggestive evidence. Hospitalization appears to be a risk factor for increased risk of esophagitis in COVID-19 patients, while it acts as a protective factor against esophageal obstruction and benign esophageal tumors. However, it may increase the risk of congenital esophageal malformations in offspring. Overall, our findings suggest that COVID-19 may cause varying degrees of damage to the esophagus through different invasive modes and the production of different cytokines. Additionally, it may unexpectedly have a protective effect on esophageal cells, indicating that the effects of COVID-19 on the esophagus are heterogeneous. For hospitalized patients, the severity of their COVID-19 is generally greater than that of COVID-19 patients who do not need to be hospitalized. Therefore, a larger amount of SARS-COV-2 is likely to invade esophageal mucosal cells via ACE2 receptors, leading to an escalating increase in the release of pro-inflammatory cytokines such as IL-1β, IL-1RA, IL-7, IL-8, IL-10, and IFN-γ, as well as the recruitment of macrophages and granulocytes from Th1 lymphocytes induced by SARS-COV-2. The levels of IL-10, IFN-γ, and the recruited macrophages and granulocytes will continue to rise, resulting in an imbalance in the ratio of Th1 to Th2 responses, which in turn triggers the release of anti-inflammatory factors such as IL-4, IL-10, and IL-13. Ultimately, this process leads to the production of a cytokine storm that exacerbates esophageal inflammation (32–34).

In COVID-19 patients, the effects of cytokines and macrophage recruitment induced by SARS-COV-2 lead to an increase in the number of macrophages in the esophageal smooth muscle. Additionally, prostaglandin E2 released from the receptor potential protein V4 (TRPV4) channel and CX3C chemokine receptor 1 (CX3CR1), expressed by macrophages, promote gastrointestinal (GI) smooth muscle peristalsis. Therefore, the substantial macrophage recruitment induced by SARS-COV-2 infection results in an increase in peristalsis of the esophageal smooth muscle, which is part of the GI smooth muscle, consequently enhancing esophageal motility and reducing the likelihood of esophageal obstruction (35), Additionally, acute inflammation of the esophagus caused by SARS-COV-2 infection of esophageal cells via the ACE2 receptor in the esophageal mucosa also accelerates the maturation of dendritic cells (DCs) and the cross-presentation of tumor antigens, initiating the activation of tumor-specific CD8+ T cells. This immune response polarizes various immune cells, including tumor-associated macrophages (TAMs), towards tumor suppression, thereby resisting the process of tumorigenesis and its progression. Consequently, this mechanism may be one of the reasons for the reduced risk of benign esophageal tumors. Thus, it provides a potential explanation for the observed decrease in the incidence of such tumors (36). Unlike esophageal obstruction and benign esophageal tumors, the risk of preeclampsia, preterm labor, and stillbirth is further elevated when the COVID-19 patient is a pregnant woman (37, 38), While esophageal atresia is among the most prominent defects observed in preterm infants with malformations, it is important to note that esophageal peristalsis is reliant on both central and peripheral vagal nerve pathways. Furthermore, the neurons located in the motor and sensory vagal nuclei of the brainstem are still in an immature state in preterm infants. This immaturity may be one of the factors contributing to the increased risk of congenital esophageal malformations (39, 40). The current treatments for COVID-19 encompass various therapeutic approaches, including nucleoside and nucleotide reverse transcriptase inhibitors, JAK1/JAK2 inhibitors, neutralizing monoclonal and polyclonal antibody therapies, as well as the administration of glucocorticosteroids such as dexamethasone and prednisone. These interventions are primarily targeted at reducing the cytokine storm induced by SARS-COV-2, thereby inhibiting the immune activation and inflammatory signaling it triggers. All of these therapeutic methods have demonstrated their ability to alleviate the clinical symptoms of COVID-19 in the respiratory system. However, further validation is needed to determine their efficacy in treating esophageal diseases (4).

Given that patients with COVID-19 present clinical symptoms of dysphagia and pancreatic achalasia, indicating a suggestive causal relationship between COVID-19 and the risk of esophageal disease that is not coincidental, we used univariate MR analysis for the first time to investigate this relationship. We fully adhered to the three assumptions required in MR analysis for instrumental variables (IVs), Considering that the IVW method is widely employed in two-sample Mendelian randomization (MR) studies and that its asymptotic standard error estimation of the causal ratio of the SNP excludes the intercept effect, enabling the exposure to offer a consistent estimate of its effect on the outcome, IVW demonstrates superior precision over MR-Egger and better accommodates between-study heterogeneity (20, 22, 41). Therefore, our MR analyses relied on IVW calculations. Notably, we found no evidence of heterogeneity for esophagitis, esophageal obstruction, benign esophageal tumors, and congenital esophageal malformations. Our study on the causality between COVID-19 and esophageal diseases was virtually unaffected by confounding and reverse causality when compared with traditional retrospective cohort studies. However, due to limitations in the available data sources, most of our studies were based on individuals of European ancestry, which may restrict the applicability of our findings to those of African and Asian ancestry. Furthermore, despite excluding confounders such as smoking, alcohol consumption, polyunsaturated fats, and inositol hexaphosphate, and employing supplementary methods like MR-Egger, Weighted, Simple mode, and Weighted mode to complement the IVW results, we cannot guarantee the complete elimination of pleiotropy. Therefore, a more extensive investigation using larger data resources is needed to delve deeper into the relationship between COVID-19 and esophageal diseases.

## 5 Conclusion

In conclusion, while the current clinical treatments for COVID-19 are primarily focused on its respiratory aspects, the potential long-term risks of esophagitis and congenital esophageal malformations should not be overlooked. Therefore, in the future, efforts should be directed towards developing a comprehensive health test for COVID-19 that encompasses multiple organs and systems. Additionally, there is a need for a more convenient viral load analysis method for SARS-CoV-2 to prevent the more serious consequences of COVID-19. These measures could help mitigate the severe outcomes of COVID-19.

## Data availability statement

Publicly available datasets were analyzed in this study. This data can be found here: summary‐level data of COVID‐19 GWAS are available at the COVID‐19 Host Genetics Initiative website (https://www.covid19hg.org/results/r7/). Data of esophageal diseases are available at the FinnGen databases (r8.finngen.fi) and GWAS Catalog (https://www.ebi.ac.uk/gwas/home).

## Ethics statement

Ethical approval was not required for the studies involving humans because summary‐level data of COVID‐19 GWAS are available at the COVID‐19 Host Genetics Initiative website (https://www.covid19hg.org/results/r7/). Data of esophageal diseases are available at the FinnGen databases (r8.finngen.fi) and GWAS Catalog. The studies were conducted in accordance with the local legislation and institutional requirements. The human samples used in this study were acquired from gifted from another research group. Written informed consent to participate in this study was not required from the participants or the participants’ legal guardians/next of kin in accordance with the national legislation and the institutional requirements.

## Author contributions

XH: Conceptualization, Data curation, Formal analysis, Investigation, Methodology, Resources, Software, Validation, Visualization, Writing – original draft. YL: Investigation, Visualization, Writing – original draft. JuL: Investigation, Visualization, Writing – original draft. GY: Writing – review & editing. XG: Writing – review & editing. GL: Formal analysis, Writing – original draft. LW: Visualization, Writing – original draft. GF: Visualization, Writing – original draft. JiL: Visualization, Writing – original draft. HZ: Funding acquisition, Project administration, Supervision, Writing – review & editing.

## Footnotes

^1^ https://www.covid19hg.org/results/r7

^2^ https://www.ebi.ac.uk/gwas/home

^3^ r8.finngen.fi

^4^ http://www.phenoscanner.medschl.cam.ac.uk/

## References

[1] Korber B Fischer WM Gnanakaran S Yoon H Theiler J Abfalterer W. Tracking changes in SARS-CoV-2 spike: evidence that D614G increases infectivity of the COVID-19 virus. Cell. (2020) 182:812–827.e19. doi: 10.1016/j.cell.2020.06.043, PMID: 32697968 PMC7332439

[2] Hu B Guo H Zhou P Shi ZL. Characteristics of SARS-CoV-2 and COVID-19. Nat Rev Microbiol. (2020) 19:141–54. doi: 10.1038/s41579-020-00459-7 33024307 PMC7537588

[3] Fung TS Liu DX. Similarities and dissimilarities of COVID-19 and other coronavirus diseases. Ann Rev Microbiol. (2021) 75:19–47. doi: 10.1146/annurev-micro-110520-023212, PMID: 33492978

[4] Yuan Y Jiao B Qu L Yang D Liu R. The development of COVID-19 treatment. Front Immunol. (2023) 14:14. doi: 10.3389/fimmu.2023.1125246 PMC9909293 36776881

[5] Taquet M Dercon Q Luciano S Geddes JR Husain M Harrison PJ. Incidence, co-occurrence, and evolution of long-COVID features: a 6-month retrospective cohort study of 273,618 survivors of COVID-19. PLoS Med. (2021) 18:e1003773. doi: 10.1371/journal.pmed.1003773, PMID: 34582441 PMC8478214

[6] Taquet M Geddes JR Husain M Luciano S Harrison PJ. 6-month neurological and psychiatric outcomes in 236 379 survivors of COVID-19: a retrospective cohort study using electronic health records. Lancet Psychiatry. (2021) 8:416–27. doi: 10.1016/S2215-0366(21)00084-5, PMID: 33836148 PMC8023694

[7] Carfì A Bernabei R Landi F. Persistent symptoms in patients after acute COVID-19. JAMA. (2020) 324:603–5. doi: 10.1001/jama.2020.12603, PMID: 32644129 PMC7349096

[8] Beydoun AS Stabenau KA Altman KW Johnston N. Cancer risk in Barrett’s esophagus: a clinical review. Int J Mol Sci. (2023) 24:6018. doi: 10.3390/ijms24076018 PMC10094310 37046992

[9] Witarto AP Witarto BS Pramudito SL Ratri LC Wairooy NAP Konstantin T. Risk factors and 26-years worldwide prevalence of endoscopic erosive esophagitis from 1997 to 2022: a meta-analysis. Sci Rep. (2023) 13:15249. doi: 10.1038/s41598-023-42636-7, PMID: 37709957 PMC10502104

[10] Rantanen TK Sihvo EIT Räsänen JV Hynninen M Salo JA. Esophageal ulcer as a cause of death: a population-based study. Digestion. (2015) 91:272–6. doi: 10.1159/000381307, PMID: 25896262

[11] Li N Yang W-L Cai M-H Chen X Zhao R Li MT. Burden of gastroesophageal reflux disease in 204 countries and territories, 1990–2019: a systematic analysis for the global burden of disease study 2019. BMC Public Health. (2023) 23:582. doi: 10.1186/s12889-023-15272-z PMC10053627 36978027

[12] Morgan E Soerjomataram I Rumgay H Coleman HG Thrift AP Vignat J. The global landscape of esophageal squamous cell carcinoma and Esophageal adenocarcinoma incidence and mortality in 2020 and projections to 2040: new estimates from Globocan 2020. Gastroenterology. (2022) 163:649–658.e2. doi: 10.1053/j.gastro.2022.05.054, PMID: 35671803

[13] Li N Zhu L Tan XY Tang HY Wang YD. Clinical and endoscopic characterization of 77 cases of benign esophageal ulcer. J Dalian Med Univ. (2020) 42:6 doi: 10.11724/jdmu.2020.06.10

[14] Dong M Zhang J Ma X Tan J Chen L Liu S. ACE2, TMPRSS2 distribution and extrapulmonary organ injury in patients with COVID-19. Biomed Pharmacother. (2020) 131:110678. doi: 10.1016/j.biopha.2020.110678, PMID: 32861070 PMC7444942

[15] Mennini M Rea F Riccardi C de Angelis P Tambucci R. SARS-COV2 and eosinophilic esophagitis: a first case. Eur J Gastroenterol Hepatol. (2021) 33:1131–2. doi: 10.1097/MEG.0000000000002109, PMID: 34213509 PMC8260336

[16] Ruz Zafra P García Sánchez CJ Pérez Ramírez A Guil Soto A Leo Carnerero E. Infection with SARS-CoV-2 as potential achalasia trigger. Rev Esp Enferm Dig. (2022) 115:203–4. doi: 10.17235/reed.2022.8975/2022, PMID: 36093995

[17] Kim J Ryu B Kim Y Choi Y Lee E. Comparative analysis of the prevalence of dysphagia in patients with mild COVID-19 and those with aspiration pneumonia alone: findings of the videofluoroscopic swallowing study. Medicina. (2023) 59:1851. doi: 10.3390/medicina59101851, PMID: 37893569 PMC10608040

[18] Angelini M Teglia F Astolfi L Casolari G Boffetta P. Decrease of cancer diagnosis during COVID-19 pandemic: a systematic review and meta-analysis. Eur J Epidemiol. (2023) 38:31–8. doi: 10.1007/s10654-022-00946-6, PMID: 36593334 PMC9807424

[19] Sanderson E Davey Smith G Windmeijer F Bowden J. An examination of multivariable mendelian randomization in the single-sample and two-sample summary data settings. Int J Epidemiol. (2019) 48:713–27. doi: 10.1093/ije/dyy262, PMID: 30535378 PMC6734942

[20] Bowden J Holmes MV. Meta-analysis and mendelian randomization: a review. Res Synth Methods. (2019) 10:486–96. doi: 10.1002/jrsm.1346, PMID: 30861319 PMC6973275

[21] Liu X du Z Wang J Wang Q Zheng Y Niu L. Association between trans fatty acids and COVID-19: a multivariate mendelian randomization study. J Med Virol. (2023) 95:e28455. doi: 10.1002/jmv.28455, PMID: 36597904

[22] Burgess S Butterworth A Thompson SG. Mendelian randomization analysis with multiple genetic variants using summarized data. Genet Epidemiol. (2013) 37:658–65. doi: 10.1002/gepi.21758, PMID: 24114802 PMC4377079

[23] Li J Bai H Qiao H du C Yao P Zhang Y. Causal effects of COVID-19 on cancer risk: a Mendelian randomization study. J Med Virol. (2023) 95:e28722. doi: 10.1002/jmv.28722 37185860

[24] Burgess S Thompson SG. Interpreting findings from Mendelian randomization using the MR-Egger method. Eur J Epidemiol. (2017) 32:377–89. doi: 10.1007/s10654-017-0255-x, PMID: 28527048 PMC5506233

[25] Bowden J Davey Smith G Haycock PC Burgess S. Consistent estimation in mendelian randomization with some invalid instruments using a weighted median estimator. Genet Epidemiol. (2016) 40:304–14. doi: 10.1002/gepi.21965, PMID: 27061298 PMC4849733

[26] Zhang H Penninger JM Li Y Zhong N Slutsky AS. Angiotensin-converting enzyme 2 (ACE2) as a SARS-CoV-2 receptor: molecular mechanisms and potential therapeutic target. Intensive Care Med. (2020) 46:586–90. doi: 10.1007/s00134-020-05985-9, PMID: 32125455 PMC7079879

[27] Sharma A Ahmad Farouk I Lal SK. COVID-19: a review on the novel coronavirus disease evolution, transmission, detection, control and prevention. Viruses. (2021) 13. doi: 10.3390/v13020202 PMC7911532 33572857

[28] Hussain M Jabeen N Raza F Shabbir S Baig AA Amanullah A. Structural variations in human ACE2 may influence its binding with SARS-CoV-2 spike protein. J Med Virol. (2020) 92:1580–6. doi: 10.1002/jmv.25832 32249956 PMC7228372

[29] Hoffmann M Kleine-Weber H Schroeder S Krüger N Herrler T Erichsen S. SARS-CoV-2 cell entry depends on ACE2 and TMPRSS2 and is blocked by a clinically proven protease inhibitor. Cell. (2020) 181:271–280.e8. doi: 10.1016/j.cell.2020.02.052, PMID: 32142651 PMC7102627

[30] Glowacka I Bertram S Müller MA Allen P Soilleux E Pfefferle S. Evidence that TMPRSS2 activates the severe acute respiratory syndrome coronavirus spike protein for membrane fusion and reduces viral control by the humoral immune response. J Virol. (2011) 85:4122–34. doi: 10.1128/JVI.02232-10, PMID: 21325420 PMC3126222

[31] Zou X Chen K Zou J Han P Hao J Han Z. Single-cell RNA-Seq data analysis on the receptor ACE2 expression reveals the potential risk of different human organs vulnerable to 2019-nCoV infection. Front Med. (2020) 14:185–92. doi: 10.1007/s11684-020-0754-0, PMID: 32170560 PMC7088738

[32] Huang C Wang Y Li X Ren L Zhao J Hu Y. Clinical features of patients infected with 2019 novel coronavirus in Wuhan, China. Lancet. (2020) 395:497–506. doi: 10.1016/S0140-6736(20)30183-5, PMID: 31986264 PMC7159299

[33] McGonagle D Sharif K O'Regan A Bridgewood C. The role of cytokines including Interleukin-6 in COVID-19 induced pneumonia and macrophage activation syndrome-like disease. Autoimmun Rev. (2020) 19:102537. doi: 10.1016/j.autrev.2020.102537, PMID: 32251717 PMC7195002

[34] Pavel AB Glickman JW Michels JR Kim-Schulze S Miller RL Guttman-Yassky E. Th2/Th1 cytokine imbalance is associated with higher COVID-19 risk mortality. Front Genet. (2021) 12:12. doi: 10.3389/fgene.2021.706902 PMC8324177 34335703

[35] Luo J Qian A Oetjen LK Yu W Yang P Feng J. TRPV4 channel Signaling in macrophages promotes gastrointestinal motility via direct effects on smooth muscle cells. Immunity. (2018) 49:107–119.e4. doi: 10.1016/j.immuni.2018.04.021, PMID: 29958798 PMC6051912

[36] Zhao H Wu L Yan G Chen Y Zhou M Wu Y. Inflammation and tumor progression: signaling pathways and targeted intervention. Therapy. (2021) 6. doi: 10.1038/s41392-021-00658-5 PMC8273155 34248142

[37] Conde-Agudelo A Romero R. SARS-CoV-2 infection during pregnancy and risk of preeclampsia: a systematic review and meta-analysis. Am J Obstet Gynecol. (2022) 226:68–89.e3. doi: 10.1016/j.ajog.2021.07.009, PMID: 34302772 PMC8294655

[38] Wei SQ Bilodeau-Bertrand M Liu S Auger N. The impact of COVID-19 on pregnancy outcomes: a systematic review and meta-analysis. Can Med Assoc J. (2021) 193:E540–8. doi: 10.1503/cmaj.202604, PMID: 33741725 PMC8084555

[39] Rittler M Campaña H Heisecke S Ratowiecki J Elias D Gimenez L. Lethality of birth defects in live born infants categorized by gestational age and birth weight. Am J Perinatol. (2023) 40:1406–12. doi: 10.1055/s-0041-1735867, PMID: 34634830

[40] Rayyan M Omari T Naulaers G Aerts R Allegaert K Rommel N. Maturation of esophageal motility and esophagogastric junction in preterm infants. Neonatology. (2020) 117:495–503. doi: 10.1159/000506481, PMID: 32208394

[41] Yavorska OO Burgess S. Mendelianrandomization: an R package for performing mendelian randomization analyses using summarized data. Int J Epidemiol. (2017) 46:1734–9. doi: 10.1093/ije/dyx034, PMID: 28398548 PMC5510723

