# Supplementary material for: The causal relationship between COVID-19 and ten esophageal diseases: a study utilizing Mendelian randomization
Source: Front Med (Lausanne). 2024 May 1;11:1346888. doi: 10.3389/fmed.2024.1346888 (PMC11094223; doi:10.3389/fmed.2024.1346888)
Supplement: Supplementary file 2 [file Data_Sheet_1.PDF]

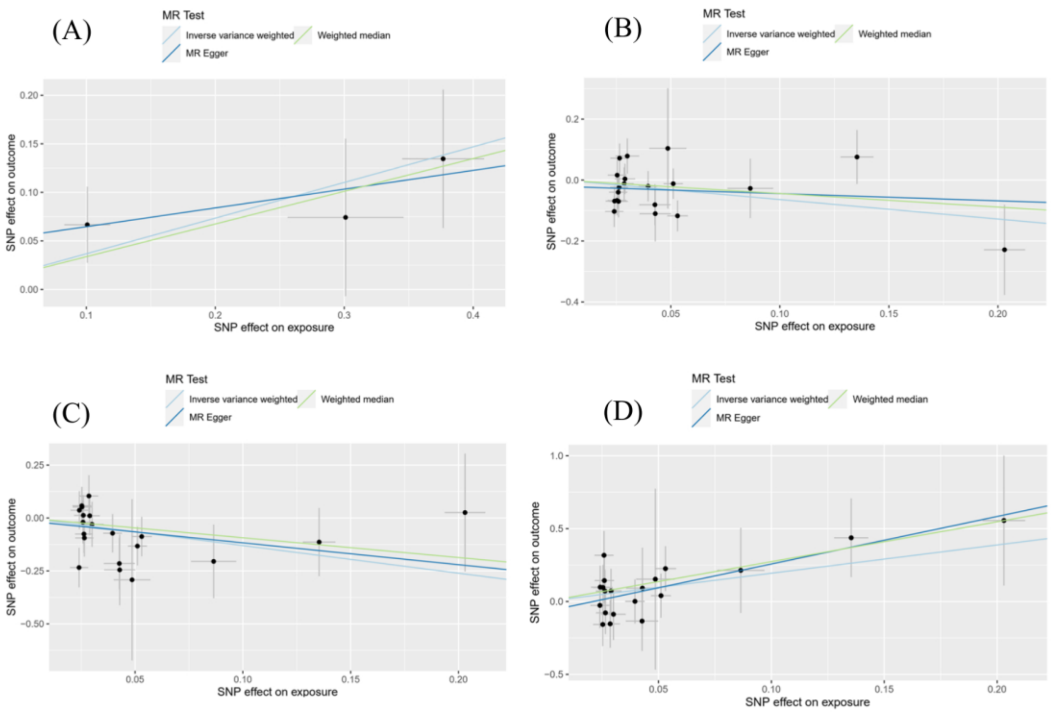

S2 Figure 1. Causal Effect Scatter Plots of COVID-19 on Esophageal Diseases. (A) Causal effect scatter plot of hospitalized COVID-19 versus non-hospitalized COVID-19 on esophagitis. (B) Causal effect scatter plot of confirmed COVID-19 on esophageal obstruction. (C) Causal effect scatter plot of confirmed COVID-19 on benign esophageal tumors. (D) Causal effect scatter plot of confirmed COVID-19 on congenital esophageal malformations.
